# Patterns of Delayed Diagnosis of Hypertension and Diabetes Among Middle-Aged and Older Adults in UK

**DOI:** 10.1093/geroni/igaf122.3399

**Published:** 2025-12-31

**Authors:** Yuming Chen, Shui-kit Cheuk, Yanran Deng, Beibei Xu

**Affiliations:** Peking University Health Science Center, Beijing, Beijing, China (People’s Republic); Peking University, Beijing, Beijing, China (People’s Republic); Peking University Health Science Center, Beijing, Beijing, China (People’s Republic); Peking University, Beijing, Beijing, China (People’s Republic)

## Abstract

Delayed diagnosis can adversely impact outcomes, leading to increased complication risks. This study aimed to investigate the influence of sociodemographic factors on diagnostic delay and associations between diagnostic delay and mortality among adults aged 37-73 years. A total of 130,843 hypertension-free individuals with abnormal blood pressure (BP) biomarker and 9,909 diabetes-free individuals with abnormal diabetes-related biomarkers, aged 37-73, from the UK Biobank, were included in two cohorts. Diagnoses were obtained through linked database, coded by ICD-10, and supplemented by self-report diagnoses or medications. Abnormal BP biomarker was defined as ≥ 140/90 mmHg; abnormal diabetes-related biomarkers included fasting glucose >7.0 mmol/L or HbA1C >48.0 mmol/mol. Logistic regression models were employed to examine the associations. During follow-up, there were 40,055 and 4,603 participants who had hypertension and diabetes diagnosis, respectively. The median diagnostic delay was 6.67 years for hypertension and 4.47 years for diabetes. Compared to individuals without cardiometabolic diseases (CMD) at baseline, those with abnormal blood pressure biomarker who had any disease of diabetes, cerebral vascular disease, and other 10 CMDs, and those with abnormal diabetes-related biomarkers who had any disease of obesity, hypertension and other 3 diseases were more likely to experience diagnostic delay. Additionally, higher levels of abnormal biomarker, older age, male, higher BMI, higher annual income, and former smoking were positively associated with diagnostic delay for both cohorts. Longer diagnostic delay, older age, higher annual income, and smoking were significantly associated with increased mortality. Baseline health conditions, age, income, and lifestyles may contribute to diagnostic delays and mortality.

